# Sedentary behaviour of pregnant women in South Africa: A cross-sectional study

**DOI:** 10.4102/safp.v67i1.6057

**Published:** 2025-05-19

**Authors:** Uchenna B. Okafor

**Affiliations:** 1 Department of Nursing Science, Faculty of Medicine and Health Sciences, Walter Sisulu University, Umthata, South Africa

**Keywords:** sedentary behaviour and prenatal physical activity, South Africa

## Abstract

**Background:**

Sedentary behaviour is a growing global public health concern that affects not only the general population but also pregnant women. Inactivity during pregnancy could have implications for the development of cardio-metabolic complications such as prenatal obesity, gestational diabetes mellitus, and hypertension, as well as mental well-being. Encouraging light prenatal physical exercise is crucial in improving maternal health of mothers as well as the baby. However, information on the sedentary behaviour of pregnant women in South Africa is limited, particularly in the Eastern Cape region. Therefore, this study investigates the proportion of time pregnant women spend in sedentary behaviours in the context of the Eastern Cape in South Africa.

**Methods:**

In this cross-sectional study, the sedentary time of 1082 pregnant women attending public health facilities in Buffalo City Municipality, Eastern Cape, South Africa, was assessed using the Pregnancy Physical Activity Questionnaire. Descriptive statistics were used to analyse the sedentary time of the participants.

**Results:**

The participants’ mean age was 27.0 years, and the standard deviation was 6.2 years. The pregnant women spent more than 3 h per day sitting (40.0%). Furthermore, a significant proportion spent 4 h to more than 6 h per day watching television or videos (46.2%) and sitting, reading, or making phone calls (51.6%) during their off-work physical activity.

**Conclusion:**

The majority of pregnant women exhibit high levels of sedentary behaviour.

**Contribution:**

Measures to encourage active physical activity during pregnancy are crucial in preventing a sedentary lifestyle among pregnant women.

## Introduction

While sedentary behaviour and time are different concepts, they are closely related. In the context of this particular study, sedentary behaviour entails an activity with a very low level of energy (≤ 1.5 metabolic equivalents [METs]) that mimics the basal metabolic rate and is devoid of a significant increase in energy expenditure.^1,2,3^ On the other hand, the latter term, sedentary time, refers to the time duration spent in this behaviour.^4^ A sedentary lifestyle, before and after pregnancy, is associated with negative maternal health and poor neonatal outcomes.^5^ Contrastingly, a decrease in sedentary behaviour or time has potential advantages for both maternal health and delivery outcomes and neonatal size.^6,7^ Therefore, prenatal and postpartum physical activity participation is crucial in promoting the desirable maternal and neonatal health outcomes associated with sedentariness among women of reproductive age. In support of this postulation, the new World Health Organization (WHO) 2020 guidelines on sedentary behaviour strongly emphasise the importance of limiting sedentary time for pregnant and postpartum women while highlighting the health benefits of replacing sedentary time with any form of physical activity.^8^ The health benefits of prenatal physical activity underscore the need to study sedentary behaviour during pregnancy because such information would be crucial to inform the development of intervention strategies to limit sedentary behaviour during pregnancy.

Understanding sedentary behaviour during pregnancy is crucial for developing effective intervention strategies. However, there is a lack of data on sedentary behaviour during pregnancy in South Africa. Pregnant women’s sedentary behaviour during pregnancy requires study because of the essential information it provides for developing intervention strategies to limit it. Motivating and supporting pregnant women to exercise during pregnancy and after require the woman’s efforts, family support, and government support. If women are informed about the benefits of physical activity during pregnancy and supported to participate in it, their attitudes towards physical activity during pregnancy may improve. The importance of prenatal physical activity in promoting health highlights the necessity of investigating sedentary behaviour during pregnancy. However, South Africa currently lacks sufficient data on this topic. Therefore, this study presents findings on sedentary behaviour during pregnancy among 1082 women in the Eastern Cape, South Africa.

## Research methods and design

The data on sedentary behaviour of pregnant women were derived from a larger project focused on developing an intervention strategy to promote prenatal physical activity in Buffalo City Municipality, Eastern Cape province, South Africa.^9^ The data collection was conducted from July 2019 to October 2019. Furthermore, it is important to mention that details regarding the methodology, population, sampling, and data collection procedures have been previously reported elsewhere.^10,11,12^ The strengthening the reporting of observational studies in epidemiology (STROBE) statement, highlighting the methodological checklist pertaining to cross-sectional studies, was adhered to.^13^

### Sedentary time measures

The Pregnant Physical Activity Questionnaire (PPAQ)^14^ assesses the sedentary behaviour and time of the participants regarding their time spent (in hours) sitting, watching television (TV), and writing, driving or riding in a car. Daily sitting, off-work computer usage or writing, driving or riding in a car, watching TV or video, and off-work sitting, reading, or calling were calculated in hours per day. More than 3 h a day sitting and using computer^15^ and more than 4 h/day offwork watching TV or video and sitting, reading, or making phone calls were used as a threshold for sedentary time.

### Data analysis

Data were analysed using descriptive statistics (frequencies and percentages) for each variable.

### Ethical considerations

Ethical approval was received from the Health Research Ethics Committee of the University of Fort Hare on 30 October 2015 with the reference number: Ref#2019=06=009=OkaforUB, and from the Department of Health. The heads of the health care facilities of Buffalo City Metropolitan Municipality also provided us permission to conduct the research. The researcher ensured confidentiality of the information from participants. The objectives and benefits of the study were explained prior to the interviews, and anonymity was ensured, as participants were not asked to record their names, but codes were used. Consent forms were given to those participants who voluntarily agreed to participate in the study. To ensure privacy, participants were interviewed in a private venue that was well ventilated with no disturbances. The right to self-determination was ensured so that participants could take part voluntarily and they could exit the study at any time without the need to provide an explanation of any sort.

## Results

The demographic characteristics of the participating women have been previously reported.^16^ Briefly, out of the 1082 participants, 75.15% were aged 19–34 years, the majority were black people (86.4%), single (66.3%), had completed Grades 7–12 (secondary education) (74.2%), and were unemployed (67.7%). Table 1 presents the participants’ daily physical activity. Most participants spent 2–3 h/day (*n* = 325; 30.0%), and more than 3 h/day sitting (*n* = 432; 40.0%), respectively. In terms of their off-work physical activity, a significant proportion spent 4 to more than 6 h/day engaged in watching TV or videos (*n* = 500; 46.2%), sitting, reading, or making telephone calls (*n* = 558; 51.6%) during their off-work physical activity (Table 2).

**TABLE 1:** Participants’ daily physical activities.

Activities	Daily physical activity frequency											
	None		< 0.5 h/day		0.5–1 h/day		1–2 h/day		2–3 h/day		> 3 h/day	
	*n*	%	*n*	%	*n*	%	*n*	%	*n*	%	*n*	%
Activity sitting	-		49	4.5	87	8.0	189	17.5	325	30.0	432	40.0
Off-work computer use or writing	777	71.8	59	5.4	103	9.5	53	4.9	32	3.0	58	5.4
Driving or riding in a car	543	50.2	257	23.8	180	16.6	51	4.7	12	1.1	39	3.6

*n*, number of participants; h/day, hours per day.

**TABLE 2:** Off-work physical activities.

Activities	Off-work activities											
	None		< 0.5 h/day		0.5–2 h/day		2–4 h/day		4–6 h/day		> 6 h/day	
	*n*	%	*n*	%	*n*	%	*n*	%	*n*	%	*n*	%
Watching television or video	70	6.5	102	9.4	36	3.3	374	34.6	110	10.2	390	36.0
Off-work sitting, reading, or making phone calls	80	7.4	250	23.1	126	11.6	68	6.3	293	27.1	265	24.5

*n*, number of participants; h/day, hours per day.

## Discussion

This cross-sectional study aimed to investigate the sedentary behaviour of 1082 pregnant women attending antenatal health facilities in the South African province of the Eastern Cape. This study confirms the high levels of sedentary behaviour among pregnant women in the Eastern Cape. While there is limited research on this topic in South Africa, an earlier study reports that, on average, pregnant women in South Africa spent 5 h daily sitting.^17^ Similarly, previous studies elsewhere have shown similar findings.^18,19,20,21^ A low level of physical activity during pregnancy has been reported among this cohort of pregnant women in South Africa,^16^ and the study confirms that most women do not meet the recommended 150 min of moderate-intensity activity per week, opting instead for light-intensity activities, such as housework.^16^ Observably, the high sedentary behaviour among pregnant women in the Eastern Cape could be attributed to a lack of facilities, resources, and awareness about physical activity and advice from health care practitioners.^16^ Furthermore, the wave of modernisation may have reduced the country’s physical activity levels, as indicated in the findings of the National Health and Nutrition Examination Survey, which reported 46% of the people being physically inactive,^22^ with pregnant women being particularly vulnerable to sedentary behaviour.^17^ Sedentary behaviour constitutes a significant global health issue, necessitating context-specific interventions to enhance physical activity during and after pregnancy.

Previous studies have alluded to the association of sedentary behaviour with various negative health outcomes, which include increased cardio-metabolic risk,^23,24^ adverse perinatal outcomes such as gestational diabetes mellitus (GDM)^25^ and obesity,^26^ hypertension, and deep vein thrombosis, emphasising the need for interventions to promote physical activity. Consequently, it is crucial to design measures to encourage women to be active during pregnancy. In this regard, a multifactorial approach can be utilised to create community awareness and develop advocacy shows or talks that provide prenatal physical activity counselling about the advantages and types of safe physical activity during pregnancy in antenatal sessions. Women should be advised to engage in light-intensity activities like walking (< 3.0 METs), which is better than no activity at all. Depending on their geographical locations, walking is a safe and accessible activity that improves pregnancy health outcomes. Studies have shown walking during pregnancy to be associated with a reduced risk of GDM,^27,28^ excessive gestational weight gain^29^ and pre-eclampsia.^29^ It is imperative to reduce sedentary time and increase physical activites to improve womenss cardio-metabolic health during pregnancy. Furthermore, the government should incorporate physical activity as a crucial element of maternal health care in South Africa regarding available policies, guidelines, counselling, and recommendations on sedentary behaviour.^30^ Judging from a public health perspective, sedentary behaviour, including that of pregnant women, is an emerging modifiable risk factor for adverse health outcomes; nonetheless, little attention is paid to pregnant women in South Africa. In this context, studies investigating the sedentary behaviour of women during pregnancy are warranted.

It is worth highlighting the limitations of this study. Notably, sedentary behaviour was self-reported; hence, there is a possibility of reporting bias, and the PPAQ’s local validity versus objective methods is lacking, which limits conclusions regarding its measurement properties, including reliability, criterion validity, construct validity, and responsiveness within the South African context.^31^ Future studies on this topic should utilise objective assessment tools, such as accelerometers, to screen for sedentary time and behaviour.

The study applied interviewer-administered questionnaires, which are more effective than self-administered questionnaires, and this could serve as a strength of this study. Also, notwithstanding the debate or lack of congruence on the recommended threshold for sedentary time, this study provides an insight regarding an important but neglected aspect of maternal health pertaining to sedentary behaviour and time during pregnancy in a large sample of pregnant women in a resource-limited setting of the Eastern Cape province, where information is lacking. Hence, the current study can serve as a basis for future research on the sedentary behaviour of women and subsequently guide intervention measures to encourage physical activity during pregnancy in the Eastern Cape.

## Conclusions

The study highlights high sedentary behaviour and time among pregnant women in the earlier-mentioned geographic context. There is a need for further research to examine factors associated with sedentary behaviour and to develop policy initiatives that would help reduce sedentary lifestyles during pregnancy.
